# Neoadjuvant Chemotherapy Alters Neuropilin-1, PlGF, and *SNAI1* Expression Levels and Predicts Breast Cancer Patients Response

**DOI:** 10.3389/fonc.2019.00323

**Published:** 2019-04-25

**Authors:** Noura Al-Zeheimi, Adviti Naik, Charles Saki Bakheit, Marwa Al Riyami, Adil Al Ajarrah, Suaad Al Badi, Khalid Al Baimani, Kamran Malik, Zamzam Al Habsi, Mansour S. Al Moundhri, Sirin A. Adham

**Affiliations:** ^1^Department of Biology, College of Science, Sultan Qaboos University, Muscat, Oman; ^2^Qatar Biomedical Research Institute, Hamad Bin Khalifa University, Doha, Qatar; ^3^Department of Mathematics and Statistics, Sultan Qaboos University, Muscat, Oman; ^4^Department of Pathology, College of Medicine, Sultan Qaboos University, Muscat, Oman; ^5^Department of Surgery, Sultan Qaboos University Hospital, Muscat, Oman; ^6^Medical Oncology Unit, Department of Medicine, College of Medicine, Sultan Qaboos University Hospital, Muscat, Oman; ^7^Department of Surgery, Wrexham Maelor Hospital, Wrexham, United Kingdom

**Keywords:** neuropilin-1, biomarker, breast, blood, response, neoadjuvant, *SNAI1*, PlGF

## Abstract

Circulating proteins hold a potential benefit as biomarkers for precision medicine. Previously, we showed that systemic levels of neuropilin-1 (NRP-1) and its associated molecules correlated with poor-prognosis breast cancer. To further identify the role of NRP-1 and its interacting molecules in correspondence with patients' response to neoadjuvant chemotherapy (NAC), we conducted a comparative study on blood and tissue samples collected from a cohort of locally advanced breast cancer patients, before and after neoadjuvant chemotherapy (NAC). From a panel of tested proteins and genes, we found that the levels of plasma NRP-1, placenta growth factor (PlGF) and immune cell expression of the transcription factor *SNAI1* before and after NAC were significantly different. Paired *t*-test analysis of 22 locally advanced breast cancer patients showed that plasma NRP-1 levels were increased significantly (*p* = 0.018) post-NAC in patients with pathological partial response (pPR). Kaplan–Meier analysis indicated that patients who received NAC cycles and their excised tumors remained with high levels of NRP-1 had a lower overall survival compared with patients whose tissue NRP-1 decreased post-NAC (log-rank *p* = 0.049). *In vitro* validation of the former result showed an increase in the secreted and cellular NRP-1 levels in resistant MDA-MB-231 cells to the most common NAC regimen Adriyamicin/cyclophosphamide+Paclitaxel (AC+PAC). In addition, NRP-1 knockdown in MDA-MB-231 cells sensitized the cells to AC and more profoundly to PAC treatment and the cells sensitivity was proportional to the expressed levels of NRP-1. Unlike NRP-1, circulating PlGF was significantly increased (*p* = 0.014) in patients with a pathological complete response (pCR). *SNAI1* expression in immune cells showed a significant increase (*p* = 0.018) in patients with pCR, consistent with its posited protective role. We conclude that increased plasma and tissue NRP-1 post-NAC correlate with pPR and shorter overall survival, respectively. These observations support the need to consider anti-NRP-1 as a potential targeted therapy for breast cancer patients who are identified with high NRP-1 levels. Meanwhile, the increase in both PlGF and *SNAI1* in pCR patients potentially suggests their antitumorigenic role in breast cancer that paves the way for further mechanistic investigation to validate their role as potential predictive markers for pCR in breast cancer.

## Introduction

Breast cancer patients with locally advanced disease are treated with preoperative cycles of neoadjuvant chemotherapy (NAC) regardless of a patients molecular subtypes (1). Patients respond differently to the preoperative chemotherapy, either completely or partially, or do not respond at all (1, 2). Factors that determine the degree of a patients' response are not yet fully understood. Therefore, molecular biomarkers and precision medicine might help to answer this question. Peripheral blood sampling is a rapid, convenient, and non-invasive method to determine an individual's pathological and physiological states. Circulating growth factors and cytokines provide a snapshot of the systemic changes in response to cancer. For instance, the circulating levels of vascular endothelial growth factor (VEGF) were shown to drive tumor survival through angiogenesis, prompting the development of the anti-angiogenic neutralizing antibody, bevacizumab (3). This has led to an improvement in patients' overall survival when combined with chemotherapy in the treatment of metastatic colon cancer (4). However, the poor efficacy of bevacizumab for the treatment of advanced breast cancer led to the withdrawal of its approval for breast cancer in 2011 by the FDA (5). A receptor closely related to VEGF is Neuropilin-1 (NRP-1), which has been associated with the progression of different types of cancer including breast cancer (6–10) and direct NRP-1 targeting via miR-376a suppressed the progression of breast cancer cells (11) Therefore, current research suggest that targeting NRP-1 might be a new strategy for cancer treatment (12). NRP-1 is a non-signaling molecule with multiple functions depending on the ligand that binds to its extracellular domain. Genentech produced an antibody that targets NRP-1, which was combined with anti-VEGF in an experimental model to show their additive antitumor activity (13). Although there are many studies confirming that NRP-1 is involved in driving tumorigenicity, clinical investigations into NRP-1 levels in patients have not been well-explored to date. Similar to NRP-1, placental growth factor (PlGF) is a member of the VEGF family and known to mediate angiogenesis, with circulating PlGF shown to be a prognostic marker for cancer. A higher plasma PlGF level was associated with progression and recurrence in colorectal cancer and oral squamous cell carcinoma (14–16). Recently, we reported the expression of NRP-1 and other associated molecules in the plasma, immune cells, and tumor tissue of a breast cancer patients' cohort and confirmed the role of NRP-1, PlGF, and *SNAI1* in breast cancer progression (17). It is well-established that NRP-1 is associated with the worst breast cancer outcomes (18). To our knowledge, there are no previous reports investigating the levels of NRP-1 in locally advanced breast cancer patients who receive NAC. Therefore, in this study, we explored the effect of NAC on the levels of plasma and tissue NRP-1 and PlGF, as well as validating their use as predictive/pharmacodynamic breast cancer biomarkers.

In this report, we are adding an extra finding, in which we show that the levels of circulating NRP-1 were significantly increased in patients who received NAC and had a partial response. Assessing patients who underwent NAC indicated that the levels of NRP-1 measured post-NAC were significantly higher in younger patients and patients with either a low or a medium body mass index (BMI), as well as in patients who remained with larger tumor size and partial response. Previously, we showed that *SNAI1* expression in the immune cells collected from the peripheral blood of breast cancer patients was significantly higher in patients with stage I disease compared with higher stages (17). In this report, we found that *SNAI1* expression in peripheral blood mononuclear cells (PBMCs) of patients who received NAC was significantly increased, especially in patients who showed a complete pathological response to the treatment, but did not increase in those who had a partial response, which indicates that *SNAI1* might be a good candidate to be used as a predictive marker for a complete pathological response. *In vitro* experiments on breast cancer MDA-MB-231 cells was done to validate the clinical observations. The knockdown of NRP-1 in the cells sensitized them to the common chemotherapy regimen (Adriyamicin/cyclophosphamide + Taxane) used in the neoadjuvant setting, which can be translated in that, patients with low levels of NRP-1 might respond better to NAC and vice versa.

## Materials and Methods

### Patients Characteristics

In a prospective setting, a cohort of 22 patients, diagnosed clinically and pathologically with locally advanced breast cancer at Sultan Qaboos University Hospital, was recruited. All 22 patients underwent NAC prior to surgery. Blood samples were collected from all 22 patients before and after the completion of NAC cycles and from 50 healthy controls. Tissue samples before (initial biopsy) and after treatment (excised tumor) were collected from all the 22 patients however, 12 out of the 22 tissue samples were only available for biomarker staining and the remaining 10 patients' tissue was not enough for research use therefore, we retrieved another 17 tissue samples from the pathology archive and were added in a retrospective setting to match the number of blood samples (22 prospective blood samples) + (12 prospective tissue + 17 retrospective tissue = 29 tissue samples). The study was approved by the ethical committee at the College of Medicine and Health Sciences, Sultan Qaboos University (License #SQU.EU/162/14, MREC#1018). Informed signed consent was obtained from all participants. All experiments were performed in accordance with institutional and national guidelines.

### Clinical Assessment, and Definition of Patients' Response Post NAC

Patients' staging and response post NAC treatment (ypTN) were classified according to American Joint Committee on Cancer (AJCC). Patients were classified as pathological complete responders (pCR) when no invasive residual carcinoma (ypT0) was identified in either the breast or lymph nodes (ypN0). The presence of *in situ* carcinoma post NAC and the absence of residual invasive disease was also categorized as pCR. Therefore, these patients were staged as either ypT0 or ypTis, respectively. Pathological partial responders (pPR) were those cases in which residual invasive cancer was present with evidence of a response to treatment. These patients would therefore have ypT stage depending on residual tumor size. Changes indicative of response to chemotherapy included fibrosis, myxoid stroma, foamy macrophages, and chronic inflammation. Further response stratification was done to determine whether the molecules in test had any association with either tumor size (ypT) or lymph node status (ypN) according to the new definition of “Perfect Pathology Report” by national cancer institute NCI publication (19).

Regarding tumor grade after NAC, and based on cancer reporting guidelines by, the College of American Pathologists, USA (20), the American Joint Committee on Cancer (AJCC) (19), and the Royal College of Pathologists, UK (21) the grade post NAC was not considered for our comparative analysis since both guidelines clearly state that for most tumors the grade remains unchanged and the prognostic significance of a change in grade post NAC has not been determined. Hence the recommendation is to grade the tumor based on the pre-treatment core biopsy.

When the patient's outcome data during follow up was compared in respect to survival, the terms remission and relapse were used. Remission is defined as the absence of cancer in laboratory tests, physical examination, and radiological imaging after the completion of the prescribed treatment. Relapse is defined as the recurrence of cancer evidenced by the former tests. Patients' characteristics at diagnosis such as age, body mass index (BMI), hormone receptor status, breast cancer subtype, chemotherapy used, overall response, tumor size, and nodal status post NAC, disease status and survival are listed in Table 1.

**Table 1:** Clinical information of breast cancer patients.

	**Blood**	**Tissue**
**PATIENTS CHARACTERISTICS**		
**Total**	22	29
**Age**		
≤50 years	86.4% (19/22)	68.9% (20/29)
>50 years	13.6% (3/22)	31.1% (9/29)
**BMI**		
18.5–24.9	22.7% (5/22)	24.2% (7/29)
25–29.9	27.3% (6/22)	13.8% (4/29)
≥30	40.9% (9/22)	58.6% (17/29)
Unknown	9.1% (2/22)	3.4% (1/29)
**Tumor size (Post NAC)**		
ypT0 (No tumor)	50% (11/22)	20.7% (6/29)
ypT1 (<2 cm)	18.2% (4/22)	31% (9/29)
ypT2 (2–5 cm)	22.7% (5/22)	20.7% (6/29)
ypT3 (>5 cm)	0% (0/22)	6.9% (2/29)
ypT4 (Tumors in the chest wall/skin or inflammatory breast cancer)	0% (0/22)	10.3% (3/29)
Not known	9.1% (2/22)	10.3% (3/29)
**Node status (Post NAC)**		
ypN0 (No nodes involved)	59.1% (13/22)	37.9% (11/29)
ypN1 (1–3 axillary lymph nodes)	22.7% (5/22)	37.9% (11/29)
ypN2 (4–9 axillary lymph nodes)	4.5% (1/22)	13.8% (4/29)
ypN3 (10 axillary lymph nodes or infraclavicular, supraclavicular or internal mammary nodes)	13.6% (3/22)	10.3% (3/29)
**Eceptor status**		
ER positive	59% (13/22)	48.2% (14/29)
ER negative	41% (9/22)	51.8% (15/29)
PR positive	54.5% (12/22)	44.8% (13/29)
PR negative	45.5% (10/22)	55.2% (16/29)
HER2 positive	45.5% (10/22)	41.3% (12/29)
HER2 negative	54.5% (12/22)	58.7% (17/29)
**Subtypes**		
Luminal A	4.5% (1/22)	13.8% (4/29)
Luminal B	18.2% (4/22)	17.2% (5/29)
Luminal B like	41% (9/22)	17.2% (5/29)
HER2 type	31.8% (7/22)	24.2% (7/29)
Triple negative	4.5% (1/22)	27.6% (8/29)
Chemotherapy^*^		
6x DCH	9.1% (2/22)	6.9% (2/29)
4xAC+4xD	40.9% (9/22)	58.6% (17/29)
4xAC+4xD+ H	13.6% (3/22)	24.2% (7/29)
3xFEC+3xD	9.1% (2/22)	3.4% (1/29)
3xFEC+3xD+ H	4.5% (1/22)	3.4% (1/29)
4xAC+12xPaclitaxel+ H (weekly)	4.5% (1/22)	3.4% (1/29)
4xAC+4xDHP	13.6% (3/22)	0% (0/29)
6xDC	4.5% (1/22)	0% (0/29)
**Response**		
pCR	36.4% (8/22)	10.34% (3/29)
pPR	59.1% (13/22)	68.98% (20/29)
Stable disease	0.0% (0/22)	10.34% (3/29)
Progression	4.5% (1/22)	10.34% (3/29)
**Disease status**		
In remission	81.8% (18/22)	65.5% (19/29)
Relapsed	18.2% (4/22)	34.5% (10/29)
**Survival**		
Survived	100% (22/22)	37.9% (11/29)
Died	0% (0/22)	24.2% (7/29)
Did not complete 3years	100% (22/22)	37.9% (11/29)

* *D, Docetaxel; H, Herceptin; A, Adriamycin; C, Cyclophosphamide; F, Fluorouracil; E, Epriubicin; P, Pertuzumab*.

### Serum Soluble Protein Detection by ELISA

Patient blood collected in EDTA-coated vacutainers was subjected to density gradient centrifugation with Histopaque (Sigma Aldrich, UK) at 400 g, with a break off for 30 min at room temperature. The separated plasma was frozen at −80°C until further analysis. The concentration of soluble NRP-1 and PlGF was measured in the plasma samples or conditioned media using ELISA kits (R&D systems, USA) according to the manufacturer's instructions. ELISA validation test was done in our previous related published article (17).

### PBMC Isolation and RNA Extraction

Anti-coagulated blood was subjected to density gradient centrifugation with Histopaque (Sigma Aldrich, UK) for 30 min at 400 g (breakoff) at room temperature. The buffy coat layer containing PBMCs was isolated, washed twice with cold PBS and pelleted at 250 g at 4°C. RNA was extracted from the PBMCs using TRI reagent (Ambion, USA), phase separation with chloroform and overnight isopropanol precipitation. The RNA pellet was washed twice in 70% ethanol in DEPC water, then dried completely and resuspended in DEPC-treated water (Ambion, USA). RNA was quantified using the NanoDrop™ 2000c spectrophotometer (Thermo Scientific, USA) and RNA quality, as indicated by the 260/280, was determined to be in the range of 1.8–2.0. One microgram of extracted RNA was treated with DNase I (Ambion, Lithuania) for 15 min at room temperature and converted to cDNA using the high-capacity reverse transcription kit (Applied Biosystems, USA). Synthesized cDNA was diluted to a final concentration of 5 ng/ul in DEPC-treated water and stored at −80°C until further analysis.

### Quantitative Real-Time PCR

Real-time PCR was conducted using the SoAdvanced mastermix (Biorad, USA). Primers were designed using the Primer Express software (Applied Biosystems, USA) and are listed in Supplementary Tables 1, 2 in our previous report (17). Fifteen nanograms of cDNA was used per reaction. The CFX96 Real-time PCR Detection System (Biorad, USA) was used under the following conditions: enzyme activation at 95°C for 20 s, 40 cycles of denaturing at 95°C for 3 s, and annealing/extension at 63.4°C for 30 s. The specificity of PCR reactions was verified by the melt curve analysis of each amplified product. Each real-time PCR reaction was performed in duplicate. A no template control (NTC) was performed for each primer pair tested in all experimental runs. Commercially available reference cDNA (Clontech, USA) was utilized as an inter-plate calibrator to identify technical variations between experimental runs. The generated Ct results were analyzed using the QBase data analysis software to generate relative expression values using the 2^−ΔΔCt^ method of calculation. The *GUSB* gene was used for normalization in qRT-PCR since it was selected according to GeNorm analysis done in our previous study (17).

### Immunohistochemistry

Immunohistochemical analysis was conducted on 29 (12 prospective+17 retrospective) pathologically confirmed locally advanced breast cancer tumors in formalin-fixed paraffin-embedded tissue. Briefly, tissue sections (3 μm) were deparaffinized using xylene and rehydrated in graded ethanol and H_2_O. Antigens were retrieved using EDTA-pH 9 solution in 95°C water bath for 30 min. Endogenous peroxidases and residual blood were blocked/removed by 2% H_2_O_2_ and the slides were washed in PBS followed by a wash in 0.05% Triton-x100 to permeabilize the cells. The tissues were blocked in 5% goat serum (Dako, USA) and incubated with primary antibody (Anti-Neuropilin 1 antibody (ab81321) or anti-PlGF antibody (ab196666) (Abcam, UK) at 4 °C overnight. Following, the slides were washed in PBS and incubated with the EnVision™ + Dual Link System-HRP (Dako, USA) labeled secondary antibody for 1 h at room temperature and followed by incubation with substrate chromogen solution (DAB) chromogen (Dako). The sections were counterstained using hematoxylin solution and dehydrated and mounted using DPX (Sigma, USA). Tissues were visualized using (NikonH600L) light microscope. Immuno Reactive Scoring (IRS) was performed for the stained slides using the following formula: IRS = SI (staining intensity) × PP (% of positive cells). Independent validation of the staining was done on normal human placenta tissue with and without primary antibody (antiNRP-1) Supplementary Figure S1.

### Cell Culture

MDA-MB-231 breast cancer cell line was purchased from Cell Lines Services CLS, Germany in 2015. The cells were maintained in monolayer cultures in 5% CO_2_ incubator at 37°C. The MDA-MB-231 cells were sustained in DMEM (Sigma, USA) supplemented with 5 mM sodium pyruvate (Sigma, USA). The cells media was supplemented with 10% fetal bovine serum (Gibco®, USA), and 2 mg/L gentamicin (Gibco, USA).

### Establishment of Resistant MDA-MB-231 Cells

The treatment modalities of MDA-MB-231 cells was done to mimic the clinical NAC treatment of breast cancer patients. Briefly, MDA-MB-231 cells were treated *in vitro* with four cycles combination of 200 nM doxorubicin (Brand name Adriamycin, Pharmacia, Italy) and 600 nM cyclophosphamide (4xAC) (Brand name Cytoxan, Baxter, Germany) followed by four cycles of 50 nM paclitaxel (4xAC+4xPAC) (Brand name Taxol, EBEWE Pharma, Austria). Each treatment cycle was 72 h long. After each cycle, the cells which remained attached were left to proliferate until confluency and the following cycle of treatment was initiated right after.

### NRP1 Knockdown Using CRISPR-Cas 9 System

CRISPR-Cas9 system was used to knockdown NRP-1 in MDA-MB-231 cells. Pre-designed NRP1 gRNA primers using the GeneArt™ CRISPR Search and Design tool (Thermo-Fisher) were used to synthesize gRNA for NRP1 knockdown (IVT-NRP1-gRNA-T2-F2: TAATACGACTCACTATAGACCAGGAGATGTAAGG and IVT-NRP1-gRNA-T2-R2: TTCTAGCTCTAAAACGGTACCTTACATCTCCTGG. The gRNA was synthesized using GeneArt™ Precision gRNA Synthesis Kit (Thermo-Fisher). The gRNA Cleanup kit (Thermo-Fisher) was used for the purification of the generated gRNA before transfection. The concentration of the purified gRNA was measured using Nanodrop (Nanodrop 2000, USA) and the gRNA band was further checked in agarose gel (100 bp). A day before transfection, 6 × 10^5^ cells were seeded in 6-well plate. GeneArt Platinum Cas9 Nuclease kit (Thermo- fisher) was used for the transfection by mixing the Cas9 Nuclease and gRNA in addition to the transfection reagent lipfectamine™ CRISPRMAX in Opti-MEM media. The mixed complex was added to transfect the cells for 48 h in 5% CO_2_ incubator at 37°C. Subsequently, the cells were diluted into 1:5, 1:10, and 1:50 to isolate the clones carrying the NRP1 knockdown. Using the colony disk isolation method, two NRP-1 knockdown clones # 15 and # 22 were selected according to the level of NRP-1 knockdown determined by western blot.

### Western Blot

NRP-1 protein expression was measured using western blotting technique. Briefly, the cells were washed with cold PBS, incubated with lysis buffer for 1–2 min (Cell Signaling technology, USA) in the presence of phenylmethylsulfonyl fluoride (PMSF) protease inhibitor (Sigma, Germany) (Sigma, Germany). Protein cell lysate was vortexed and centrifuged for 20 min at 4°C. The supernatants were collected, and protein quantification was done using Pierce bicinchoninic acid (BCA) protein assay kit (Thermo Fisher). Protein lysate samples were all adjusted to have 100 μg of total protein per sample and were electrophoretically separated using 7.5% sodium dodecyl sulfate polyacrylamide gel electrophoresis and then transferred onto polyvinylidene difluoride membranes (BioRad, USA). The immunoblots were then blocked with 5% non-fat milk and subsequently probed with rabbit primary monoclonal antibodies, NRP-1 (Ab Cam, UK Catalog #ab81321) or GAPDH as a normalizing internal control (cell signaling technologies catalog #2118) at a 1:1,000 dilution, incubated at 4°C overnight. The blots were then washed three times for 5 min with PBS and incubated with goat anti-rabbit IgG horseradish peroxidase conjugated secondary antibody at 1:5,000 dilutions (Abcam, UK) for 2 h at room temperature and developed using the clarity western ECL substrate (BioRad, USA). The densitometric analysis of the protein bands was performed using the Image lab software (BioRad, USA).

### Colony Formation Assay

The ability of the MDA-MB-231 cells parental or NRP-1 Knockdown variants to form colonies before and after treatment was tested. The cells were treated with combination of both 200 nM Adriyamicin (doxorubicin) and 600 nM cyclophosphamide or 50 nM paclitaxel [IC_50_ (22)] for 72 h. Then, the cells were seeded at a density of 1,000 cells/well in 6-well plates (Corning, USA) and incubated for 14 days at 37°C in 5% CO_2_ incubator and the media was changed twice during this period. After the 2 weeks of incubation, the colonies were washed with PBS and stained with 25% crystal violet/methanol solution for 15 min at room temperature. Crystal violet stain was removed, and the wells were washed with tap water.

### Statistical Analysis

The paired *t*-test was used as a gold standard test for the differential expression of plasma or tissue proteins before and after NAC. The Shapiro–Wilk test for normality was conducted to determine the distribution of breast cancer patients in the cohort studied. While the distribution of the plasma protein dependent variables was confirmed to be normal, the PBMC gene expression data indicated non-normal distribution. Considering the heterogeneity in the population distribution and the unequal and limited number of cases among the subgroups studied, the PBMC gene expression data set was log_10_-transformed to attain normality in the population distribution. The results that were significant using the paired *t*-test were further tested by univariate analysis to compare the measured values in patient samples, before and after treatment, to the levels measured in the healthy controls used in our previous study, with Tukey used as a *post-hoc* test. The Kaplan–Meier curve was generated to calculate the overall survival of patients regarding the low or high immunoreactive score (IRS) for NRP-1 tissue expression and the log-rank test (Mantel-Cox) was used to indicate significance. Associations where *p* < 0.05 were considered significant (95% confidence interval). Poorly represented subgroups (*n* < 3) were excluded from the analysis to avoid interpretation errors. The IBM SPSS software (Version 22) was used for all statistical analyses and graph preparations.

## Results

### Plasma NRP-1 Levels Are Induced by Chemotherapy and Correlate With a Poor Response in Patients

**Figure 1:**
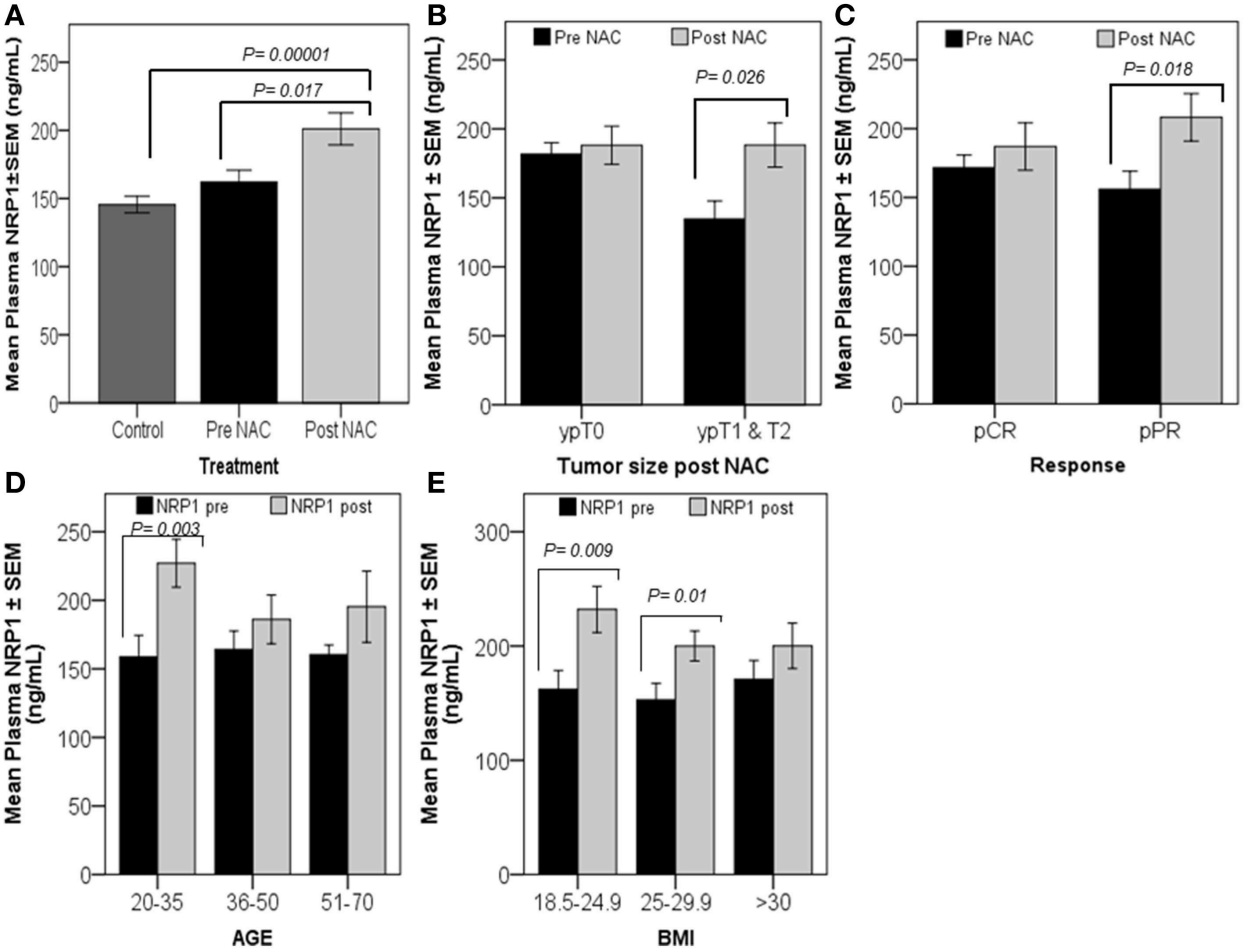
Significant increase in plasma NRP-1, post-NAC, was correlated with poor response. Univariate analysis **(A)** showed a significant increase in plasma NRP-1, post-NAC, compared to the healthy controls and pre-NAC levels. The graphs **(B–E)** represent the paired *t*-test for the mean plasma NRP1 levels ± SEM. The graphs reveal a significant increase in plasma NRP1, post-NAC, in patients who had tumors, ypT1&2 **(B)**, and in those with partial response (pPR) **(C)**. NRP-1 levels were found to increase in younger patients and patients with either low or moderate BMI **(D,E)**. *p* ≤ 0.05 is considered to indicate statistical significance.

The univariate analysis comparing plasma NRP-1 levels in healthy controls (*n* = 50), patients with locally advanced disease prior to the initiation of NAC (pre-NAC) (*n* = 22), and post-treated patients (post-NAC) (*n* = 22) indicated that there was a significant increase in plasma NRP-1 levels in post-treated patients, compared to their initial level (*p* = 0.017) and the level of the healthy normal controls (*p* = 0.00001) (Figure 1A). The paired *t*-test was used to determine the differential levels of plasma NRP-1 pre- and post-NAC. The analysis of data indicated that the levels of plasma NRP-1 were increased significantly (*p* = 0.026) in patients who remained with a large tumor size (*n* = 9, ypT1&2) and partially responded to the treatment (*p* = 0.018) (*n* = 13, pPR) (Figures 1B,C). However, no significant change was observed in the NRP1 level in patients who had no tumor post NAC (*n* = 11, ypT0) or in patients who showed complete response to NAC (*n* = 8, pCR). In addition, NRP-1 plasma levels, post-NAC, were significantly higher (*p* = 0.003) in young patients (*n* = 6, 20–35 years) and in cases with a low or a medium BMI [(*n* = 5, BMI 18.5–24.9 (*p* = 0.009) and *n* = 6, BMI 25–29.9 (*p* = 0.01)] (Figures 1D,E).

### Low Tissue NRP-1 Expression Post-NAC Is Correlated With Improved Survival

**Figure 2:**
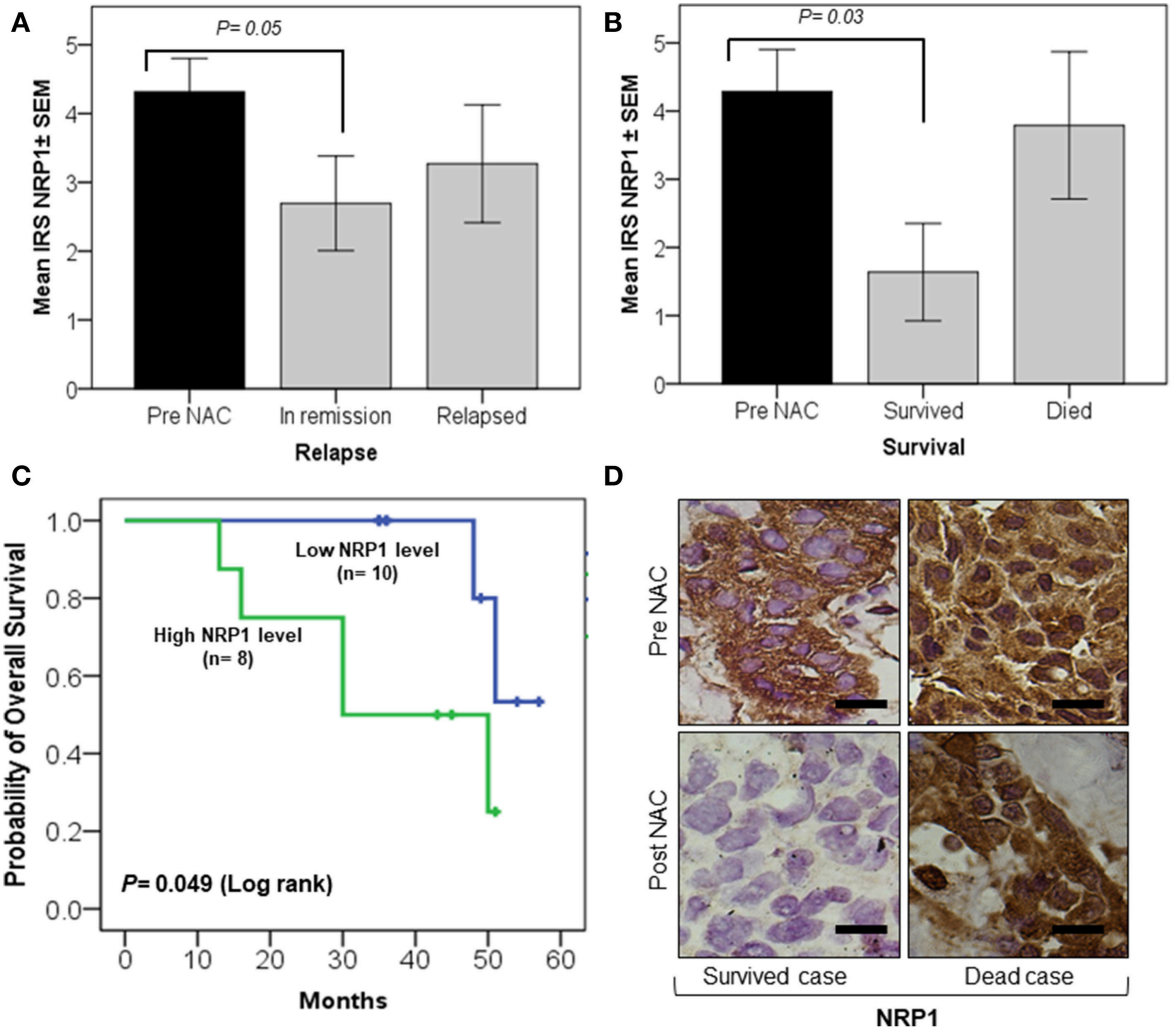
Significant decrease in tissue NRP-1 expression, post-NAC, is linked with better overall patient survival. The graphs **(A,B)** represent the univariate analysis of NRP1 ± SEM IRS values in breast cancer tissues pre- and post-NAC. A significant decline in NRP-1 expression, post-NAC, was observed in the tumor tissue of patients under remission **(A)** and who survived **(B)**. The Kaplan–Meier curve **(C)** shows a significant improvement in overall survival in patients whose tumors were represented with a low IRS tissue NRP-1 score after NAC. The representative images of immunohistochemistry staining **(D)** showed a decrease in tissue NRP-1 levels as a result of chemotherapy in surviving vs. non-surviving individuals, scale bar = 50 μm. *p* ≤ 0.05 is considered to indicate statistical significance.

Univariate analysis showed that tissue NRP-1 expression was reduced in post-NAC specimens (*p* = 0.05) of patients under remission (*n* = 19), while there were no changes in relapsed patients (*n* = 10) (Figure 2A). Similarly, tissue NRP-1 expression, post-NAC, was significantly decreased (*p* = 0.03) in all surviving patients (*n* = 11), but not in patients who died (*n* = 7) (Figure 2B). A Kaplan–Meier graph indicated that patients who received NAC and remained with high tissue NRP-1 levels (*n* = 8) had lower overall survival compared with patients whose tissue NRP-1 decreased post-NAC (*n* = 10), (log-rank *p* = 0.049) (Figure 2C). Representative immunohistochemistry images for tissue NRP-1 expression indicated a dramatic decrease in tissue NRP-1 levels, post-NAC, in the patients who survived, compared to those who died (Figure 2D).

### Plasma Levels of PlGF Were Increased in Pathological Complete Responders

**Figure 3:**
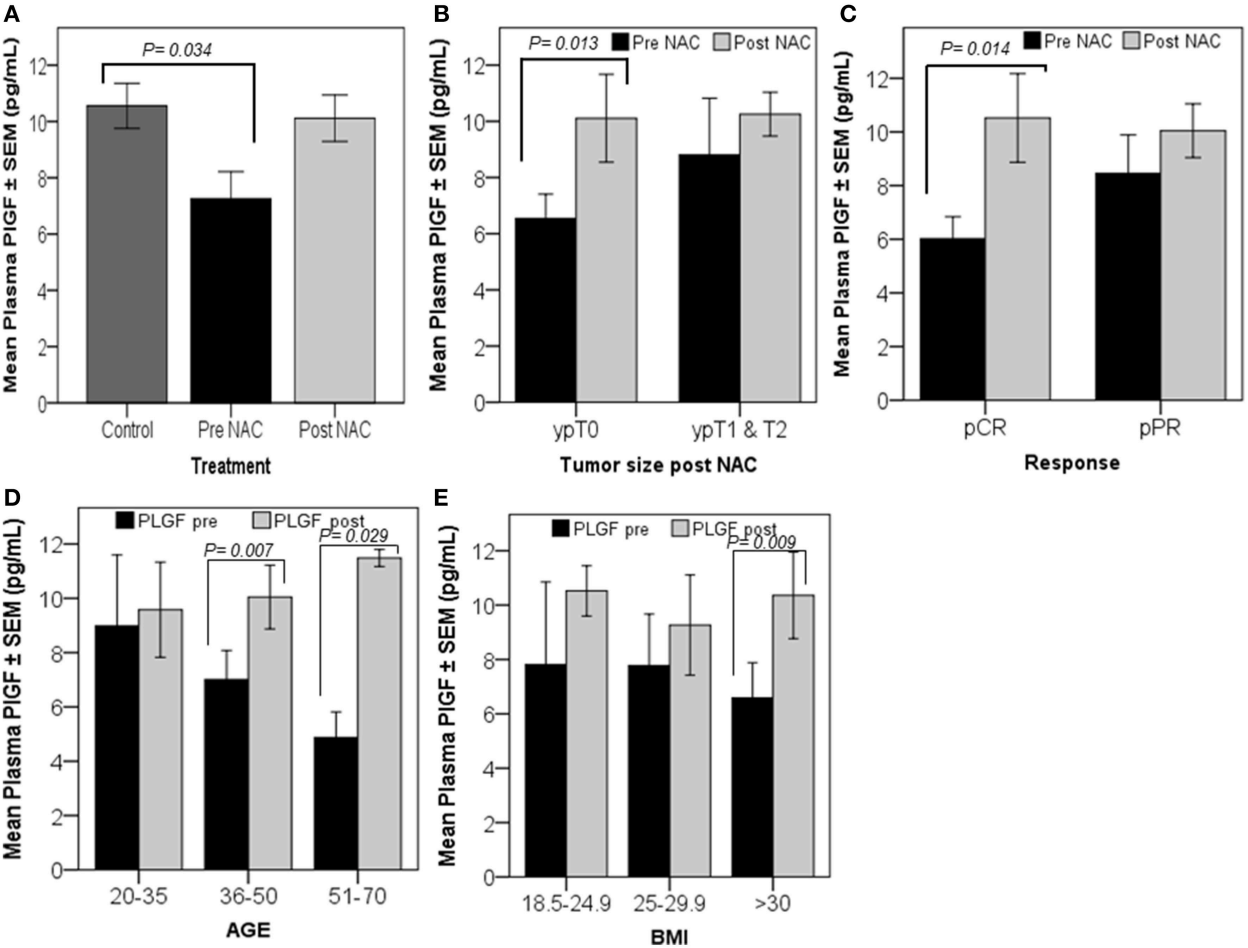
Patients with high-circulating PlGF levels, post-NAC, showed a better response. Univariate analysis revealed a significant decrease in plasma PlGF levels in breast cancer patients measured prior to the initiation of chemotherapy (pre-NAC) compared to the levels measured in healthy controls **(A)**. The graphs **(B–E)** represent the paired *t*-test results, which show the mean plasma PlGF ± SEM. A significant increase in the PlGF level was detected in patients who were tumor-free after finishing NAC cycles, ypT0 **(B)**, and in patients who responded completely to the treatment, pCR **(C)**. The graphs **(D,E)** show PlGF levels increased significantly post-NAC in patients aged between 36–50 and 51–70 years, and in patients who have a BMI >30. *p* ≤ 0.05 is considered to indicate statistical significance.

Univariate analysis indicated that the basal levels of plasma PlGF, pre-NAC (*n* = 22), were significantly lower (*p* = 0.034) than the levels found in healthy controls (*n* = 50) (Figure 3A). The paired *t*-test indicated a relative increase in plasma levels of PlGF in patients who showed complete tumor regression (*n* = 11, ypT0) (*p* = 0.013) and a pathological complete response (*n* = 8, pCR) (*p* = 0.014) after NAC (Figures 3B,C). Increased plasma PlGF was observed in older and patients [*n* = 13, 36–50 years (*p* = 0.007) and *n* = 3, 50–71(*p* = 0.029)] and in patients with a high BMI [*n* = 9, BMI >30(*p* = 0.009)] (Figures 3D,E).

### *SNAI1* Expression in PBMCs Is Upregulated in Complete Responders

**Figure 4:**
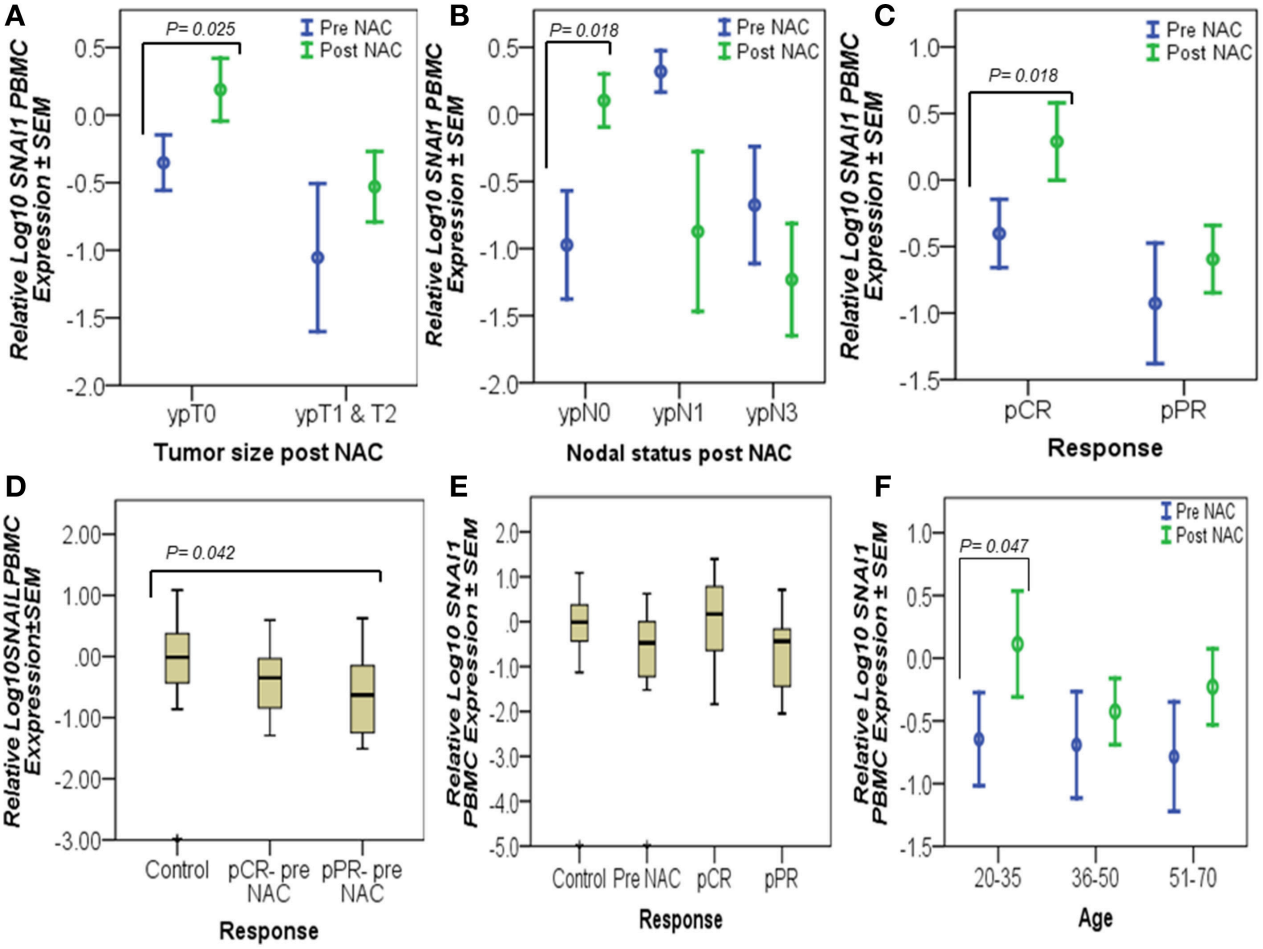
Complete responders had significantly high *SNAI1* expression in their PBMCs. The graphs represent the relative Log_10_ PBMC *SNAI1* ± SEM in controls and breast cancer patients, pre- and post-NAC, as measured by RT-qPCR. An upregulated level of *SNAI1*, post-NAC, was detected in patients who had no residual tumors (ypT0), no lymph node involvement (ypN0), and in patients with a complete response (pCR), as tested by the paired *t*-test **(A–C)**. Similarly, univariate analysis **(D,E)** showed a significant decrease in the pre-NAC expression of *SNAI1* in pPR patients, compared to the expression in the healthy controls **(D)** and an increased trend in *SNAI1* expression, post-NAC, in pCR patients almost at the levels measured in the healthy controls **(E)**. However, *SNAI1* expression declined in breast cancer patients when measured prior to the start of NAC (pre-NAC) and in those who did not respond to treatment (pPR) **(E)**. In addition, a significant increase in *SNAI1* expression was detected in the PBMCs of young patients as a result of NAC using paired *t*-test **(F)**. *p* ≤ 0.05 is considered to indicate statistical significance.

*SNAI1* levels measured in patients PBMCs indicated that this transcription factor is significantly increased in patients who had no residual tumor (*n* = 11, ypT0) (*p* = 0.025) or diseased lymph nodes (*n* = 13, ypN0) after NAC and in pathological complete responders (*n* = 8, pCR) (*p* = 0.018) (Figures 4A–C). Univariate analysis showed a significant decrease (*p* = 0.042) in the initial expression (pre-NAC) of *SNAI1* in patients who showed a partial response (*n* = 13, pPR) to NAC, compared to the expression in the healthy controls (*n* = 50) (Figure 4D). Additionally, a trend of increased *SNAI1* expression post-NAC was observed in patients with pCR (*n* = 8) similar to the levels detected in the healthy controls (Figure 4E). *SNAI1* expression in PBMCs was significantly increased in young patients (*n* = 6, 20–35 years old) (*p* = 0.047) (Figure 4F).

### Chemotherapy Treatment for MDA-MB-231 Cells Increased NRP-1 Expression Levels

**Figure 5:**
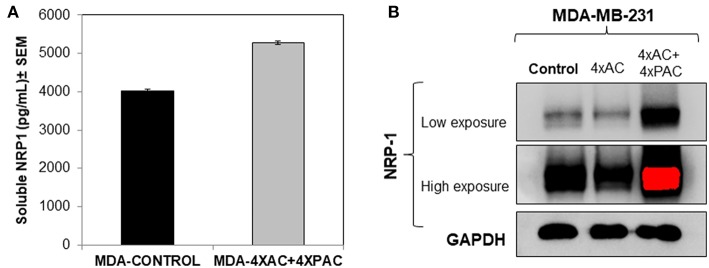
*In vitro* cellular analysis show that resistant MDA-MB-231 cells, treated with four cycles of Adriamycin and cyclophosphamide and four cycles of Paclitaxel (mimicking the clinical treatment setting of breast cancer), caused an increase in the levels of soluble **(A)** and cellular NRP-1 **(B)** detected by ELISA and western blot, respectively.

The resistant MDA-MB-231 cells to chemotherapies 4xAC+4xPAC expressed higher levels of soluble NRP-1 in the conditioned media and exhibited increased levels of cellular NRP-1 as represented by the increase in the intensity of the protein band on western blot (Figures 5A,B).

### Neuropilin-1 Knockdown Decreased the Number of Colonies Formed by MDA-MB-231 Cells and Sensitized Them to Chemotherapy

**Figure 6:**
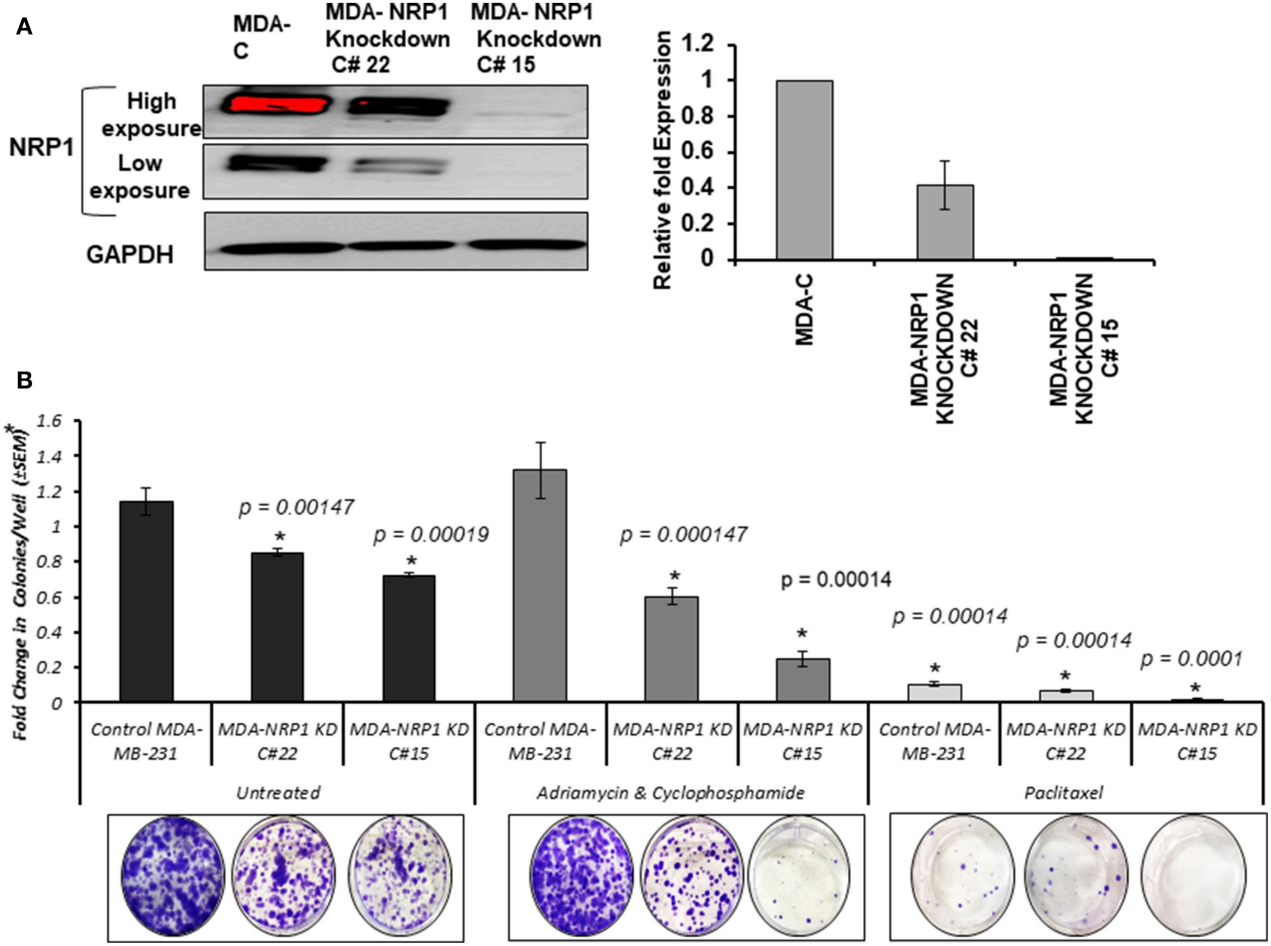
NRP-1 knockdown reduced the colony formation ability of MDA-MB-231 cells and sensitized the cells to chemotherapy. **(A)** Western blot analysis showed the successful knockdown of NRP-1 to almost 58% in the isolated clone MDA- NRP-1 Knockdown Clone#22, and 99% in MDA-NRP-1 knockdown Clone#15. GAPDH was used for protein normalization. The graph represents the densitometry quantification of the mean ± standard error for the relative fold change in three independent experimental replicates and *p* < 0.05 was considered the cut of value for significance. **(B)** The bar graph represents the average (±SEM) number of colonies from three independent replicas presented as the fold change in respect to the parental MDA-MB-231 untreated cells (MDA-C). The NRP-1 knockdown on its own reduced the ability of the MDA-MB-231 cells to form colonies. However, treatment with Adriamycin and Cyclophosphamide did not affect the control parental MDA-MB-231 cells, but it did decrease the number of colonies formed by the two NRP-1 knockdown cells. In the case of Paclitaxel treatment, the number of colonies were decreased in control parental MDA-MB-231 and the 58% NRP-1 knockdown cells (MDA- NRP1 knockdown C# 22) but totally inhibited the colony formation in the 99% NRP-1 knockdown cells (MDA- NRP1 knockdown C# 15). Asterisks indicate significantly different values from control untreated MDA-MD-231 cells (*p* < 0.05).

NRP-1 was efficiently knocked down in MDA-MB-231 cells using CRISPR Cas-9 as described in the materials and methods section. Two different clone variants; MDA- NRP-1 knockdown clone # 22 (58% knockdown) and MDA- NRP-1 knockdown clone # 15 (99% knockdown) were isolated (Figure 6). The quantification of NRP-1 expression was determined by measuring the density of the expressed bands as represented in the graph from three independent experiments (Figure 6). Clonogenic assay showed that NRP-1 knockdown caused a reduction in the ability of the cells to form colonies. Treating NRP-1 knockdown variants with combination of Adriamycin and cyclophosphamide (AC) or paclitaxel differentially reduced the ability of the cells to form colonies, however. Treating control parental MDA-MB-231 cells with AC didn't not affect the formation of the colonies only paclitaxel reduced its clonogenic ability.

## Discussion

A major obstacle of cancer management is drug resistance. Breast cancer patients with locally advanced breast cancer receive NAC to reduce tumor size, which makes it easier to be excised by surgery (12). The progression of the disease, post-NAC, is usually due to the presence of innate chemoresistant cells or acquired resistance throughout the cyclic treatment (23). In this study, we analyzed the plasma and PBMCs of breast cancer patients prior to the start of NAC and post-NAC. We also investigated the differential expression of proteins and genes that we have previously shown to be involved in poor-prognosis breast cancer cases (17); however, their role in predicting a response to chemotherapy has not been studied before. Plasma NRP-1, post-NAC, was upregulated in patients who were classified as partial responders to NAC. More importantly, the patients who died had high levels of tissue NRP-1, post-NAC, than surviving patients. This notion is interesting, since it is in concordance with previous findings in non-small lung cancer (NSCLC), where patients who had a high expression of NRP-1 after chemotherapy had shorter disease-free and overall survival (24). Another study indicated that overexpressing NRP-1 caused chemoresistance in pancreatic cancer cells through the MAPK signaling pathway (25), Similarly, we observed an increase in the expression levels of soluble and focal NRP-1 using ELISA and western blot, respectively, in an MDA-MB-231 breast cancer cell-chemoresistant model (4xAC+4xPAC) generated in our lab (Figures 5A,B). In addition, we recently reported that NRP-1 overexpression was induced by a combination of Adriamycin and cyclophosphamide-treated BT474 breast cancer cells (26). While the inhibition of NRP-1 increased chemosensitivity for different kinds of cancer cells (27). More recently, a study showed that the inhibition of NRP-1, using a small molecule antagonist, caused a combined reduction in angiogenic and tumorigenic ability (28). In line with the previous findings, the *in vitro* knockdown of NRP-1 in MDA-MB-231 cells in this study supports the fact that NRP-1 high expression leads to more resistance to chemotherapy similar to a recent report which indicated the role of NRP-1 promoting resistance to oncogene targeted therapies (29). Therefore, our result confirms the usefulness of the strategy to target NRP-1 in combination with chemotherapy in patients with a partial response to NAC alone, which thus determines that NRP-1 is a pharmacodynamic biomarker in breast cancer.

Although we did not find any significance using Kaplan–Meier analysis between PlGF plasma or tissue expression, before and after chemotherapy, with patients' survival, the plasma levels were significantly high in complete responders (pCR). A similar increase in plasma PlGF was reported as a result of anti-angiogenic treatment (30); however, there are no reports on PlGF levels after NAC. Although we reported earlier that plasma PlGF levels were not significantly different from those in healthy controls (17), in this study, we showed that there was a significant decrease in pre-NAC plasma PlGF, when compared with healthy controls. This discrepancy arises from differential study design criteria, since our previous report was conducted on breast cancer patients, regardless of their disease stage or treatment plan; whereas, in this study, we only focused on those patients who presented with locally advanced disease and underwent NAC. A previous study showed the prognostic value of PlGF in patient tissue toward breast cancer progression (31), which is consistent with our previous findings that tissue PlGF is higher in metastatic breast cancer, compared with locally advanced breast cancer patients (17). In this study, we indicate that plasma PlGF increases significantly post-NAC in complete responders, thereby suggesting its potential use as a pharmacodynamic biomarker for breast cancer post NAC, similar to an earlier study in renal cancer, which described PlGF as a pharmacodynamic biomarker for anti-VEGF therapy (32).

In addition to PlGF, the overexpression of *SNAI1* in PBMCs, post-NAC in complete responders (no residual tumor and no nodal disease), points to its protective role in breast cancer prognosis. The expression of *SNAI1* in complete responders (pCR) attained a similar level in the healthy controls. A previous report showed that the *SNAI1* protein product, Snail, was expressed at lower levels in breast tumor tissue compared with normal breast tissue (33). We reported a similar finding in PBMCs from breast cancer patients, who have significantly lower *SNAI1* expression compared with healthy controls (17).

## Summary and Conclusions

We conclude that NRP-1 expression in breast tumor tissue, post-NAC, is a potential predictive biomarker for breast cancer survival. Circulating plasma PlGF levels are lower in locally advanced breast cancer patients compared to healthy individuals, while they increased, post-NAC, in patients who responded completely to the treatment. *SNAI1* expression in immune cells are downregulated in breast cancer patients and increased, similar to healthy control levels in complete responders to NAC, indicating their potential protective role in breast cancer. All the studied molecules thus serve as good candidates for breast cancer prognosis and targeted treatment. Overall, the main aim of this study was to understand grossly the relationship between patients' response to NAC treatment regardless of the regimen used and potential molecular biomarkers but does not compare variables between the different chemotherapy treatment types, since treatment can't be always the exact same for each patient and depends on individual disease stage, subtype and sensitivity to drugs. And finally, the results of knocking down NRP-1 in MDA-MB-231 cells indicated that the cells became more sensitive to the treatment regardless of the drug used. Therefore, the *in vitro* results were consistent with the clinical observations in which patients with low levels of NRP-1, responded much better than those who remained with high levels of NRP-1. The exploratory results obtained from such a small sample size study are still interesting for future validation on larger scale clinical research studies.

## Author Contributions

NA-Z: methodology formal analysis, investigation, software, writing—review and editing. AN: data curation, methodology, software, writing—review and editing. CB: methodology formal analysis, software, writing-review and editing. MA: methodology formal analysis-review and editing. AA: data curation, investigation. SA and KA: methodology formal analysis. KM: methodology, writing-review and editing. ZA: methodology and data curation. MSA: conceptualization, methodology, investigation, writing-review and editing. SAA: conceptualization, supervision, investigation, writing–original draft, writing– review and editing, and visualization.

### Conflict of Interest Statement

The authors declare that the research was conducted in the absence of any commercial or financial relationships that could be construed as a potential conflict of interest.
