# Supplementary material for: Neoadjuvant Chemotherapy Alters Neuropilin-1, PlGF, and SNAI1 Expression Levels and Predicts Breast Cancer Patients Response
Source: Front Oncol. 2019 Apr 25;9:323. doi: 10.3389/fonc.2019.00323 (PMC6494932; doi:10.3389/fonc.2019.00323)
Supplement: Supplementary file 1 [file Image_1.pdf]

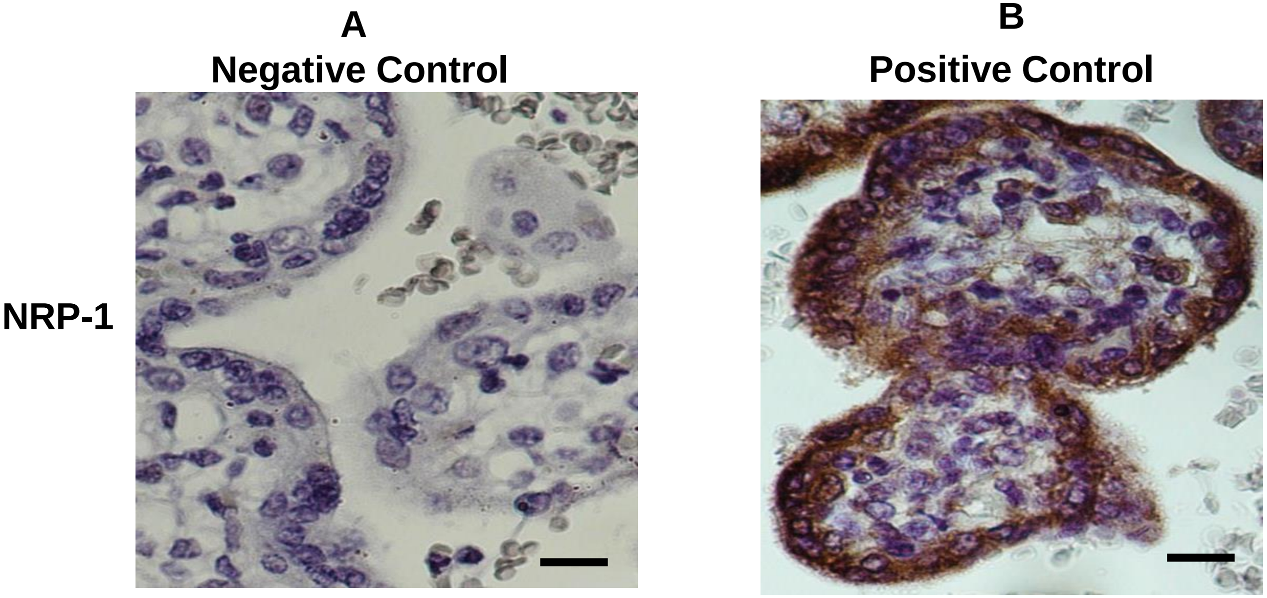

Supplementary Figure S1. Immunohistochemistry staining for normal human placental tissue as a positive control for NRP-1. Control placental tissue was simultaneously stained with breast tumor tissue to ensure the specificity of the staining. A) control negative staining for placental tissue were the NRP-1 primary antibody (Abcam Cat#: ab81321) was not added to the sections serving as a back ground for staining. B) Full staining with NRP-1 antibody respectively, staining show positive accumulation of the protein in the placental tissue. Scale bar=50 µm
